# *In Vitro* Efficacy of Aqueous and Methanol Extract of *Cassia siamea* Against the Motility of *Caenorhabditis elegans*

**DOI:** 10.21315/tlsr2020.31.3.10

**Published:** 2020-10-15

**Authors:** Haladu Ali Gagman, Nik Ahmad Irwan Izzauddin Nik Him, Hamdan Ahmad, Shaida Fariza Sulaiman, Rahmad Zakaria, Farah Haziqah Meor Termizi

**Affiliations:** 1 School of Biological Sciences, Universiti Sains Malaysia, 11800 USM Pulau Pinang, Malaysia; 2 Department of Biological Sciences, Faculty of Science, Bauchi State University Gadau, 751 Itas Gadau, Nigeria

**Keywords:** Ethnoveterinary, *C. elegans*, Anthelmintic, Inhibition, Motility, Ethnoveterinari, *C. elegans*, Anthelmintik, Perencatan Motiliti

## Abstract

Gastrointestinal nematode infections can cause great losses in revenue due to decrease livestock production and animal death. The use of anthelmintic to control gastrointestinal nematode put a selection pressure on nematode populations which led to emergence of anthelmintic resistance. Because of that, this study was carried out to investigate the efficacy of aqueous and methanol extract of *Cassia siamea* against the motility of *C. elegans* Bristol N2 and *C. elegans* DA1316. *Caenorhabditis elegans* Bristol N2 is a susceptible strain and *C. elegans* DA1316 is an ivermectin resistant strain. *In vitro* bioassay of various concentrations of (0.2, 0.6, 0.8, 1.0 and 2.0 mg mL^−1^) aqueous and methanol extracts of *C. siamea* was conducted against the motility of L4 larvae of *C. elegans* Bristol N2 and *C. elegans* DA1316. The L4 larvae were treated with 0.02 μg mL^−1^ of ivermectin served as positive control while those in M9 solution served as negative control. The activity of the extracts was observed after 24 h and 48 h. A significant difference was recorded in the extract performance compared to control at (*P* < 0.001) after 48 h against the motility of the larvae of both strains. The methanol extracts inhibited the motility of *C. elegans* Bristol N2 by 86.7% as well as DA1316 up to 84.9% at 2.0 mg mL^−1^ after 48 h. The methanol extract was more efficient than aqueous extract (*P* < 0.05) against the motility of both strains of *C. elegans. Cassia siamea* may be used as a natural source of lead compounds for the development of alternative anthelmintic against parasitic nematodes as well ivermectin resistant strains of nematodes.

## Abstract (ms)

Jangkitan nematod gastrousus boleh menyebabkan kerugian besar hasil disebabkan oleh pengurangan pengeluaran dan kematian haiwan. Penggunaan antelmintik untuk kawalan nematod gastrousus telah menyebabkan tekanan pilihan yang menjurus kepada kemunculan kerintangan antelmintik. Oleh sebab itu, kajian ini dijalankan untuk mengkaji keberkesanan ekstrak akueus dan metanol *Cassia siamea* terhadap motiliti *C. elegans* Bristol N2 dan *C. elegans* DA1316. *Caenorhabditis elegans* Bristol N2 merupakan strain rentang dan *C. elegans* DA1316 merupakan strain rintang ivermektin. Bioasai *in vitro* kepelbagaian kepekatan (0.2, 0.6, 0.8, 1.0 dan 2.0 mg mL^−1^) ekstrak akueus dan ekstrak metanol *C. siamea* dijalankan terhadap motiliti L4 *C. elegans* Bristol N2 dan *C. elegans* DA1316. Larva L4 yang dirawat dengan 0.02 μg mL^−1^ ivermektin dijadikan sebagai kawalan positif manakala, larutan M9 dijadikan sebagai kawalan negatif. Aktiviti ekstrak itu diperhatikan selepas 24 jam dan 48 jam. Perbezaan yang signifikan dicatatkan pada prestasi ekstrak berbanding dengan kawalan pada (*P* < 0.001) selepas 48 jam terhadap motiliti larva bagi kedua strain. Ekstrak metanol merencat motiliti larva *C. elegans* Bristol N2 86.7% dan *C. elegans* DA1316 sehingga 84.9% selepas 48 jam pada kepekatan 2.0 mg mL^−1^. Ekstrak metanol lebih berkesan daripada ekstrak akueus (*P* < 0.05) terhadap motiliti kedua-dua jenis *C. elegans. Cassia siamea* boleh digunakan sebagai sumber semula jadi sebatian utama untuk pembangunan anthelmin alternatif terhadap nematod parasit dan juga nematod rintang ivermektin.

**Highlights**

- The barks extracts show some promising results after been exposed at 24 h and 48 h to methanol and aqueous extracts of *C. siamea*.
- The worms were paralysed, and become non-active and at the higher concentration, it kills the worms.
- The first ever report demonstrating the use of *Cassia siamea* against *C. elegans* expands the knowledge on the usage of *C. siamea* and indirectly can reduced the usage of the drug for the treatment of the nematodes.

## INTRODUCTION

*Caenorhabditis elegans* is a free-living none parasitic nematode organism that naturally inhabits the soil of the temperate region. It is easily maintained in the laboratory because of its advantage of the short life cycle (Kumarasingha *et al*. 2014). The quest for a multicellular model organism with few cells and easy to reproduce for an experiment in the embryonic developmental studies drew the attention of researchers to explore *C. elegans* in the 60s (Katiki *et al*. 2011). For decades, *C. elegans* has been used as a major tool for biomedical research and currently been employed by several researchers as an invaluable model for drugs discovery and development (Katiki *et al*. 2011).

Therefore, *C. elegans* is one of the most studied model organisms to overcome the obstacles interfering with the screening of products for alternative anthelmintic. For instance, *in vivo* screening of plant as an alternative to synthetic anthelmintic is faced with numerous challenges ranging from the high cost of *in vivo* screening and time-consuming procedures to ethical constraints. Furthermore, difficulty in obtaining suitable adult parasitic nematode that could be used to evaluate the efficacy of anthelmintic makes it difficult to study drugs effect on the adult parasites *in vitro*. These challenges have been overcome with the discovery of *C. elegans* and its subsequent uses as a laboratory model in screening for anthelmintic. The application of *C. elegans* as a model in the screening of plants and plant products for anthelmintic have generated the advantages of cheap *in vitro* activities, easy maintenance, short generation time coupled with the transparent body of *C. elegans* which may permit easy observation of the drug’s effects against the adult organism. In addition, it is possible to study the drugs effect or activity of compounds against adult *C. elegans* and applied the idea of the effect of the drug on parasitic nematodes of the same clades (Kumarasingha *et al*. 2014).

Furthermore, sustainable worm control has been threatened by anthelmintic resistant species of the parasites especially those of small ruminants because understanding the genetic basis of resistant of parasitic nematode is extremely difficult. This problem has been overcome to some extent with the emergence of *C. elegans* as a research model especially in studying anthelmintic resistance. This organism has been central to define the mechanism of anthelminthic action and this could serve as a model to solve the problem resulting from anthelmintic resistant (Holden-Dye & Walker 2014).

*Caenorhabditis elegans* belongs to the same Clade V with some economically influential nematodes parasites from the family of Trichostrongylidae such as *Haemonchus contortus, Trichostrongylus colubriformis, Ostertagia ostertagi* and *Oesophagostomum columbianum*, as they share common anatomical and physiological relationship with *C. elegans*. According to Jones *et al*. (2005), by being a parasite makes it difficult to commence the study in the laboratory without using the artificial laboratory animal hosts. This is because parasitic nematodes are difficult to culture and analyse independently of their hosts (Blaxter *et al*. 1998). In addition, there are some limitation to study the parasite in their natural hosts which is including the labour-intensive, time-consuming, issues on animal ethic and the costs (Wimmersberger *et al*. 2013). Because of that, this study is focused on the use of two strains of *C. elegans. Caenorhabditis elegans* N2 strain is a wild type strain that susceptible to all anthelmintic and *C. elegans* DA1316 is an ivermectin-resistant strain which contains mutation in *avr-14, avr-15* and *glc-1*. Each of genes encode glutamate-gated chloride channel and highly resistant to ivermectin (Dent *et al*. 2000). It is reasonably assumed that drugs effect on the *C. elegans* could be reproduced on such parasitic nematodes (Kumarasingha *et al*. 2014).

*Caenorhabditis elegans* has been used for more than 30 years for the study on anthelmintic resistant and screening for the potential compound or drug target in parasitic nematodes (Simpkin & Coles 1981). An analysis of the *Annona crassiflora* (marolo) extract on *C. elegans* shows some promising results where it inhibited the motility of the tested worms up to 98.13% (Machado *et al*. 2015). In addition, treatment of *C. elegans* in *Combretum mucronatum* (bushwillow) was found to be effective to reduce the survival rate up to 89.2% (Agyare *et al*. 2014). Study on *C. elegans* CB193 levamisole resistant strain shows that, *Psidium guajava* (guajava) extract inhibit the motility and oviposition activity of the worms which suggested some potential anthelmintic activity of the plant. A similar evaluation of activities of others plant extracts were carried out with different plant extract show similar results on the motility of *C. elegans* (Phiri *et al*. 2014; Barnett *et al*. 2016; Kong *et al*. 2014).

Looking into potential use of *C. elegans*, this study focused on the use of *Cassia siamea* (yellow cassia) plant extract. *Cassia siamea* is an evergreen medium size tree which can grow with a straight trunk to the height of 18 m and with a trunk girth of 30 cm in diameter and belongs to the family of Fabiaceae (Kamagaté *et al*. 2014). The plant grows naturally in many Asian countries such as Malaysia, Brunei and China. It is extensively cultivated in afforestation programmes in many African countries (Orwa *et al*. 2009). *Cassia siamea* has been reported to be a useful antioxidant, antimicrobial, antimalarial, anticancer, antihypotensive, anti-inflammatory and antidepressant (Ntandou *et al*. 2010). The objective of this study was to investigate the efficacy of aqueous and methanol extract of *C. siamea* against the motility of *C. elegans* Bristol N2 (susceptible to ivermectin) and *C. elegans* DA1316 (ivermectin resistant strain).

## MATERIALS AND METHODS

### Collection of Plant Materials

Stem barks of *C. siamea* were collected among the trees in the savannah scattered vegetation of Azare, in Katagum Local Government located between latitude 11° 40′ 35″ N and longitude 10° 11′ 41″ E, Nigeria. *Cassia siamea* with the voucher specimen number 900078 was authenticated and deposited at the Department of Biological Sciences herbarium of Bauchi State University Gadau, Nigeria.

### Phytochemical Extraction

Powdered plant material was subjected to extraction by maceration in water (aqueous) and methanol according to the method explained by Lienou *et al*. (2015). The extraction was carried out in the School of Biological Sciences, Universiti Sains Malaysia.

#### Aqueous extraction

The aqueous extraction was carried out by soaking 50 g of the dry powdered sample in 250 mL of distilled water in a beaker (1:5 w/v). The setup was kept for 5 days at room temperature in the range between 25°C–28°C. The supernatant was filtered using Whatman no.1 filter paper before it was evaporated to dryness in an oven at 45°C for 1 week to obtain the dry extract. The dry extract was stored at 4°C in a labeled sterile glass vial before use (Lienou *et al*. 2015). This same procedure was applied for methanol extract where 80% methanol was used as a solvent.

The formula described by Zhang *et al*. (2007) was used to compute the percentage yields of dry aqueous and methanol extract of *C. siamea* as follows:

$$\text{Percentage yield of extract }(\%)=\frac{\text{weight of the dry extract}}{\text{weight of the original sample}}\times 100$$

### Phytochemical Screening of Plant Extracts for Secondary Metabolites

This was carried out by mixing of various reagents and plant extract and resulted to reactions indicated by colour changes that were used to identify the secondary metabolites in aqueous and methanol extracts based on the methods of Maobe *et al*. (2013) and Gaziano *et al*. (2015) as follows: Alkaloids (Dragendorff’s reagents and 2 m of H_2_SO_4_), flavonoids (2% ammonia solution + 2% NaOH + 2% HCl), saponins (froth formation on shaking with water), Salkowski’s test for steroids (acetic anhydrite + 2% + H_2_SO_4_) phenols (using 2% FeCl_3_), Tannins (2% FeCl_3_), terpenoids (Chloroform + H_2_SO_4_).

### Phytochemical Analysis

This assay was aimed at determining the total tannins and total phenolics content of aqueous and methanol extracts of *C. siamea*. Total tannins content was determined by Folin-Denis spectrometric method as described by Oliveira *et al*. (2009). A solution of 1 mg mL^−1^ concentration of the desired extract of *C. siamea* was prepared by dissolving in 50% (v/v) methanol. Exactly 20 μL of the extract and tannic acid (standard) of serial concentrations (0, 0.5, 1, 1.5, 2, 2.5, 3, 3.5 mg mL^−1^) were separately mixed with 100 μL of 25% Folin-Denis reagent and kept for 3–5 min. 80 μl of Na_2_Co_3_ was added and the setup in 96 well plates was incubated in darkness for 1 hr. Calibration curve of absorbance values against the varying concentration of the tannic acid standard was plotted and the regression equation (y = ax + b) was obtained with the aid of Microsoft Excel Software version 2016. The tannins content of the samples was finally calculated in Tannic Acid Equivalent (TAE mg^−1^). The same procedure was used for determination of total phenolic content. However, Gallic acid with the concentrations of (0, 0.5, 1, 1.5, 2, 2.5, 3.3.5 mg mL^−1^) was used as standard instead of tannic acid and Folin-Ciocalteau reagents were used instead of Folin- Denis reagent. Phenolic content was calculated in mg Gallic Acid Equivalence (GAE mg^−1^).

### Maintenance of *C. elegans* and Synchronisation

*Caenorhabditis elegans* Bristol N2 and *C. elegans* DA1316 were supplied by *C. elegans* Genetic Center (CGC) USA. Synchronised populations of *C. elegans* was used for this investigation as recommended by Baugh (2013). The synchronised population of the *C. elegans* was obtained by adding 5 mL of fresh alkaline bleaching solution (a mixture of 1N NaOH and hypochlorite in the ratio of 1:2) to 1 mL of a mixture of gravid adults and eggs from old culture plates in a 15 mL centrifuge tube. The content was mixed by shaking for about 5 min until most of the bacteria and the adult worms were dissolved. The bleaching process was stopped by the addition of 8 mL of M9 solution (3 g KH_2_PO, 6 g Na_2_HPO_2_, 6 g NaCl and 1 M Mg SO_4_ in 1 L of distilled water) to the content. Centrifugation of the content was carried out at 1500 rpm and the clear supernatant was aspirated. The pelleted content was re-suspended in M9 solution shaken and the centrifugation was repeated. The eggs obtained were transferred to a fresh Nematodes Growth Medium (NGM) plate (mixtures of 3 g NaCl 17 g agar and 2.5 g of Bacto peptone in 1000 mL of distilled water autoclaved and 1 m IM CaCl_2_, 1 mL 5 mg mL^−1^ cholesterol, 1 mL 1 M MgSO_4_, 25 mL 1M KPO_4_ buffer, all autoclaved except for cholesterol were added). The plate was incubated in an incubator shaker at 20°C overnight before the L1 larvae were observed after 24 h (Baugh 2013). The L1 larvae were transferred to a new NGM plate seeded with *E. coli* OP50 and incubated over night at 2°C and the L4 larvae observed after about 30 h (Radman *et al*. 2013).

#### In vitro bioassay of crude methanol and aqueous extracts of C. siamea against the motility of C. elegans Bristol N2 and C. elegans DA1316 L4 larvae

Evaluation of the efficacy of the extracts was based on the standard of the World Association for the Advancement of Veterinary Parasitology (WAAVP). The standard considers the ovicidal or larvicidal efficacy of the anthelmintic agent to be effective when it is up to 90% but moderately effective when it is lower than 90% but up to 80%. The concentration of 2.0 mg mL^−1^ stock of the required extracts of *C. siamea* was obtained by dissolving 200 mg of the crude extract in 10 mL of 1% Tween 80 solution and was diluted with 90 mL of M9 buffer. The stock solution was further diluted with M9 solution to give a serial concentration of 0.2, 0.6, 0.8, 1.0 and 2.0 mg mL^−1^ according to the method of Kumarasingha *et al*. (2014). An ivermectin solution of 0.02 μg mL^−1^ was prepared by dissolving 1 mg solid sample of ivermectin in 1 mL of 1% DMSO before dilution with an M9 solution to obtained 0.02 μg mL^−1^.

Approximately 100 L4 larvae of the required strain of *C. elegans* in a suspension of 50 μL were added to each of the 24 wells. The amount of 1 mL of each serial concentration (0.2, 0.6, 0.8, 1.0 and 2.0 mg mL^−1^) of the required extract was added to the larvae in the wells with three replicates for each concentration. Larvae were treated with 0.02 μg mL^−1^ of ivermectin with three replicates served as a positive control whereas those in three wells treated with M9 solution served as negative control. The setup was incubated at 20°C; counting of motile larvae after 24 h and 48 h was done under an inverted microscope. Three independent experiment with both methanol and aqueous extract on each strain of *C. elegans* was carried out and the average results were recorded. The percentage of worm motility (%WM) was calculated using the formula of Tariq *et al*. (2009) as follows:

$$\text{WM}\%=\frac{\text{number of worms in negative control well}-\text{number of mobile worms in treatment well}}{\text{number of worms in negative control well}}\times 100$$

## STATISTICAL ANALYSIS

The data were presented as the mean ± SE and computed using Microsoft® Excel 2016 software. Statistical analysis was carried out using IBM SPSS^®^ version 24. Kolmogorov-Smirnov was used to investigate the normality of data. The percentage motility inhibitory efficacy among different concentrations of the extracts and the negative control was compared using one-way ANOVA. The post hoc statistical significance used was least square difference (LSD) and the difference between the means was considered significant at *P* < 0.05. Probit analysis was applied to calculate the inhibitory concentration (IC_50_). The efficacies of aqueous and methanol extracts were compared using paired sample *t*-test.

## RESULTS

### Extract Yields

The percentage yield of 6.0% was recorded for aqueous extracts whereas 7.31% was recorded for methanol extract.

### Phytochemical Screening

More varieties of secondary metabolites were revealed in the methanol extract than in the aqueous extract of *C. siamea*. Alkaloids, saponins, tannins and phenols were confirmed in aqueous extract. Screening of the methanol extract revealed tannin, alkaloids, saponins, terpenoid flavonoid and phenols (Table 1).

### Total Phenolic and Total Tannins Content

The methanol extract of *C. siamea* recorded a total tannins content of 1.92 TAE mg^−1^, higher than the aqueous extract which recorded the tannins content of 0.68 TAE mg^−1^. Similarly, the total phenolics content recorded by the methanol and aqueous extracts of *C. siamea* were 49.53 GAE mL^−1^ and 29.03 GAE mL^−1^, respectively (Table 2).

### Results of *in vitro* Bioassay of Crude Methanol and Aqueous Extracts Against the Motility of L4 Larvae of *C. elegans* Bristol N2 and *C. elegans* DA1316

The results revealed increased in the efficacy of both methanol and aqueous extracts as the concentrations of extracts and time increased (Table 3). This was observed as the lowest performance was recorded at the lowest concentration of 0.2 mg mL^−1^ at 24 h. This is indicated by the highest larval motility of 91.8% (8.2% inhibited) and 81.4% (18.6% inhibited) recorded in the aqueous and methanol extracts respectively against *C. elegans* Bristol N2 at the lowest concentration of 0.2 mg mL^−1^ at 24 h. Similarly, the motility of 93.8% (6.2% inhibited) and 83.6% (16.4% inhibited) were recorded against the motility of *C. elegans* DA1316 by aqueous and methanol extracts respectively at the lowest concentration of 0.2 mg mL^−1^ after 24 h (Table 3).

The highest efficacy was recorded at the highest concentration of 2.0 mg mL^−1^ after 48 h where a highly significant difference between the treatment and control at *P* < 0.001 was recorded against the motility of both strains of *C. elegans*. At the concentration of 2.0 mg mL^−1^ after 48 h, the methanol extracts of *C. siamea* was effective against the motility of both strains of *C. elegans*. This was evidence as only 13.3% (86.7% inhibited) of *C. elegans* Bristol N2 were motile whereas 15.1% (84.9% inhibited) of *C. elegans* DA1316 where motile at the concentration of 2.0 mg mL^−1^ after 48 h. The aqueous extract, on the other hand, was ineffective at the concentration of 2.0 mg mL^−1^ after 48 h. However, the aqueous extract exhibited an appreciable level of biological activities as up to 30.6% motility of *C. elegans* Bristol N2 and 33.9% for *C. elegans* DA1316 was recorded at 2.0 mg mL^−1^ after 48 h (Table 3).

The IC_50_ (50% inhibitory concentration) assumed a similar trend to that of percentage motility. Based on the IC_50,_ the methanol extract was more efficient than the aqueous extract (*P* < 0.05). This was evidenced by the lower IC_50_ value of 0.766 mg mL^−1^ recorded by methanol extract compared to a higher IC_50_ value of 1.295 mg mL^−1^ recorded by aqueous extract against *C. elegans* Bristol N2 after 48 h (Table 4). Similarly, the methanol extract which exhibited a lower IC_50_ value of 0.906 mg mL^−1^ proved more promising against the motility of *C. elegans* DA1316 after 48 h than the aqueous extract which recorded a higher IC_50_ value of 1.45 mg mL^−1^ of *C. elegans* DA1316 (Table 4).

Generally, a significant difference was recorded in the larvicidal activity between aqueous and methanol extract (*P* < 0.05) against both strains of *C. elegans*. However, the extracts performance against the motility of *C. elegans* Bristol N2 and *C. elegans* DA1316 were statistically the same (*P* > 0.05). A highly significant difference between the performance of ivermectin and the extracts against *C. elegans* DA1316 was recorded (*P* < 0.001) as ivermectin was not effective against *C. elegans* DA1316. This is also evidenced by the performance of ivermectin and the negative control against *C. elegans* DA1316 were statistically the same (*P* > 0.05). However, ivermectin proved more efficient than the plant extracts (*P* < 0.05) against the motility of *C. elegans* Bristol N2.

## DISCUSSION

This work was aimed at investigating the efficacy of aqueous and methanol extract of *C. siamea* against the motility of *C. elegans* Bristol N2 and *C. elegans* DA1316. The high percentage larval motility in the negative control throughout the assay coupled with the variation in the percentage larval motility among the different concentrations of the extracts was a clear indication that the diluent used in the preparation of the extract solution did not interfere with the anthelmintic properties of the extracts.

Presently, there are scarce scientific records on the anthelmintic activity of *C. siamea* extracts against *C. elegans*. Results in this report suggested that the potential use of the *C. siamea* plant extract as an alternative for the treatment of nematode resistant worm (*C. elegans* DA1316). *Caenorhabditis elegans* DA1316 is an ivermectin resistant strain. Incubation with the extract not only effect the motility of the worms but also killed them *in vitro* after 24 h and 48 h treatment. Significant difference occurred in the larvicidal efficacy between the positive control (ivermectin) and all the treatment with the various concentrations of both methanol and aqueous extracts at *P* < 0.001 against *C. elegans* DA1316. The methanol extract of *C. siamea* was effective against the larval motility of *C. elegans* Bristol N2 and *C. elegans* DA1316 after 48 h. The same phenotypes were observed for *C. elegans* N2 treated with ivermectin compared to *C. elegans* DA1316 that treated with the plant extracts. Both strains tested were paralysed and were inhibited with both treatments.

Results in this study also indicated that the methanol plant extract of *C. siamea* is more lethal compared to the aqueous extract of *C. siamea* against *C. elegans* DA1316 at all concentrations. According to Ahmed *et al*. (2014), the effectiveness of plant extracts depends on the phyto-constituent composition and type of solvent chosen for the extraction. This can be explained by the present of different level of secondary metabolites in the different extracts. Also, phenolic and tannins contents were higher in methanol extract compared to aqueous extract (*P* < 0.001). The variation in the types and quantity of secondary metabolites between the aqueous and methanol extract might be attributed to the difference in the polarity of the two solvents. Water extracts only polar compounds because it is purely a polar solvent whereas methanol which is a polar as well as none polar solvent and extracted both polar and none polar compounds. The polar and none polar characteristic of methanol enables it to extract more varieties of secondary metabolites than water as suggested by Dailey and Vuong (2015). In addition, the nonpolar characteristic of methanol also enables it to dissolve the nonpolar cell walls of the plants to release more quantity and varieties of secondary metabolites (Tiwari *et al*. 2011). The presence of saponins, tannins, flavonoids, and other phenolic compounds in the extracts of *C. siamea* might have been responsible for its anthelmintic activity based on the previous reports on the anthelmintic roles of these compounds. For instance, tannins interfere with energy phosphorylation inhibited in the nematodes thereby leading to energy depletion and starvation of the nematode and might eventually lead to paralysis and death of the nematode. Tannins also bind to the free protein of the nematode to form a complex as well as other structures in the nematode such as cuticle, digestive, reproductive tract thereby interfering with their normal functions. Tannins might also inhibit egg hatch and larval development (Debiage *et al*. 2016). Whereas saponin increase pore formation and permeability of the cell membrane of the nematode causing vacuolization and disintegration of the nematode’s integument (Wang *et al*. 2010). Previous study in bacterial also suggested that in which tannins and flavonoids usually form complex with bacterial cell, bind with protein and may inhibit the enzyme resulting in kill of bacteria (Aiello *et al*. 2003).

The anthelmintic activity of *C. siamea* observed in this study is similar to the anthelmintic activites of extracts from plants such as crude extracts of *Kaya senegalensis*, *Annona senegalensis* and *Annogeisus leiocarpus* which were reported to be ovicidal and larvicidal against the adult of *C. elegans* (Ndjonka *et al*. 2014). Ndjonka *et al*. (2011) also reported strong anthelimintic activity exhibited by ellagic acid and gentistic acid from *Anogeissus leiocarpus* against *C. elegans* DA1316 among other commercial drugs resistant strains of *C. elegans* tested. Piña-Vázquez *et al*. (2017) also reported effective anthelmintic activity of aqueous extract of *Psidium guajava* againts the motility of levamisole resistant strain of *C. elegans* CB193. The effectiveness of *C. siamea* also has been reported against several strains of bacteria in previous study (Phaiphan *et al*. 2014). The antibacterial potential was tested by disc diffusion method against seven strains of bacteria, *Staphylococcus* sp. BCC 5357, *Bacillus cereus* ATCC 33019, *Vibrio parahaemolyticus* ATCC 17802, *Escherichia coli* ATCC 25922, *Salmonella typhimurium* ATCC 14028, *Salmonella enteritidis* ATCC 13076 and *Pseudomonas aeruginosa* BCC 30506. In that study, the extract of *C. siamea* leaves were inhibited the Gram-positive strains more than the Gramnegative strains. Kamagaté *et al*. (2014) reported that *C. siamea* could be useful as antimicrobial, antibacterial, anticancer among several other medicinal values. It has been used in Asia and other tropical countries as medicinal herbs against ringworm, anthelmintic and anti-rheumatic (Singh *et al*. 2013).

## CONCLUSION

The different extracts of *C. siamea* were studied for their secondary metabolites, total phenolic and total flavonoid contents in addition to anthelmintic potential against *C. elegans* DA1316 an ivermectin-resistant strain. Both extracts showed significant anthelmintic activities in all different concentrations. Methanol extract exhibited highest anthelmintic activity as well as more phenolic and tannins contents in comparison with the aqueous extract. Therefore, the results obtained support that *C. siamea* as potential source of anthelmintic compounds and activities that could be used for an alternative for the anthelmintic treatment. Further research work will focus on the isolation and characterisation of secondary metabolites responsible for anthelmintic activities.

## Figures and Tables

**Table 1:** Phytochemical screening of *Cassia siamea*.

Secondary metabolites	Aqueous	Methanol
Alkaloids	−	+
Saponins	+	+
Tannins	+	+
Terpenoids	−	+
Steroids	−	−
Flavonoids	−	−
Phenols	+	+

*Note:* + Presence of metabolite; − Absence of metabolites

**Table 2:** Total phenolics and total tannins contents of *Cassia siamea*.

Extract	Phenolic contents (GAE mg^−1^ ± SD)	Tannin contents (TAE mg^−1^ ± SD)
Aqueous	29.03 ± 0.72	0.68 ± 0.38
Methanol	49.53 ± 1.81	1.92 ± 0.08

**Table 3:** The efficacy of *Cassia siamea* extracts against the motility of *C. elegans* Bristol N2 and *C. elegans* DA1316 L4 larvae.

Concentration (mg mL^−1^)	*C. elegans* Bristol N2		*C. elegans* DA1316	

	Time/hour			

	24	48	24	48
Aqueous extract				
0.2	91.8 ± 0.58	81.0 ± 0.58	93.8 ± 0.58	84.2 ± 0.56
0.4	87.9 ± 0.65	78.2 ± 0.58	90.9 ± 0.70	81.2 ± 0.47
0.6	84.6 ± 0.88	73.1 ± 0.72	88.2 ± 0.58	75.2 ± 0.55
0.8	80.6 ± 0.87	65.2 ± 0.55	84.2 ± 0.53	69.2 ± 0.58
1.0	77.1 ± 0.60	56.2 ± 0.58	79.3 ± 0.54	62.2 ± 0.55
2.0	69.5 ± 0.67	30.6 ± 0.68	74.8 ± 0.60	33.9 ± 0.67
Ivermectin	36.5 ± 0.89	2.33 ± 0.47	98.5 ± 0.27	96.4 ± 0.29
M9 solution	97.9 ± 0.28	96.3 ± 0.71	98.3 ± 0.74	97.2 ± 0.03

Methanol extract				
0.2	81.4 ± 0.58	72.6 ± 0.58	83.6 ± 0.58	73.9 ± 0.35
0.4	77.4 ± 0.58	67.5 ± 0.65	79.2 ± 0.57	67.9 ± 0.67
0.6	74.2 ± 0.61	62.9 ± 0.73	75.9 ± 0.65	64.2 ± 0.61
0.8	71.3 ± 0.58	45.2 ± 0.61	70.6 ± 0.63	55.3 ± 0.55
1.0	65.9 ± 0.65	30.8 ± 0.65	67.2 ± 0.61	43.2 ± 0.51
2.0	53.9 ± 0.65	13.3 ± 0.98	58.1 ± 0.57	15.1 ± 0.73
Ivermectin	37.2 ± 0.61	2.67 ± 0.72	98.3 ± 0.58	95.8 ± 0.26
M9 solution	98.0 ± 0.45	96.1 ± 0.35	97.1 ± 0.82	96.1 ± 0.32

*Notes*: Data are presented as a percentage means ± standard error for 3 independent experiments. L4 larvae of *C. elegans* Bristol N2 and DA1316 were incubated for 48 h in different concentration of the extracts (0.2, 0.4, 0.6, 0.8, 1.0 and 2.0 mg mL^−1^). Counting of the immotile worms was carried out after 24 h and 48 h.

**Table 4:** The IC_50_ of aqueous and methanol extracts of *Cassia siamea* against *C. elegans* Bristol N2 and *C. elegans* DA1316 after 48 h.

Extract type	*C. elegans* Bristol N2	*C. elegans* DA1316

	IC_50_ mg mL^−1^	IC_50_mg mL^−1^
Aqueous	1.295	1.450
Methanolic	0.766	0.906
